# Exploring Barriers to and Enablers of the Adoption of Information and Communication Technology for the Care of Older Adults With Chronic Diseases: Scoping Review

**DOI:** 10.2196/25251

**Published:** 2022-01-07

**Authors:** Sojib Bin Zaman, Raihan Kabir Khan, Roger G Evans, Amanda G Thrift, Ralph Maddison, Sheikh Mohammed Shariful Islam

**Affiliations:** 1 Department of Medicine School of Clinical Sciences at Monash Health Monash University Melbourne Australia; 2 Department of Health Sciences James Madison University Harrisonburg, VA United States; 3 Cardiovascular Disease Program Biomedicine Discovery Institute Monash University Melbourne Australia; 4 Department of Physiology Faculty of Medicine, Nursing and Health Sciences Monash University Melbourne Australia; 5 Institute for Physical Activity and Nutrition School of Exercise & Nutrition Sciences Deakin University Geelong Australia

**Keywords:** older adults, gerontechnology, usability challenges, chronic disease, information technology, mobile phone

## Abstract

**Background:**

Information and communication technology (ICT) offers considerable potential for supporting older adults in managing their health, including chronic diseases. However, there are mixed opinions about the benefits and effectiveness of ICT interventions for older adults with chronic diseases.

**Objective:**

We aim to map the use of ICT interventions in health care and identified barriers to and enablers of its use among older adults with chronic disease.

**Methods:**

A scoping review was conducted using 5 databases (Ovid MEDLINE, Embase, Scopus, PsycINFO, and ProQuest) to identify eligible articles from January 2000 to July 2020. Publications incorporating the use of ICT interventions, otherwise known as eHealth, such as mobile health, telehealth and telemedicine, decision support systems, electronic health records, and remote monitoring in people aged ≥55 years with chronic diseases were included. We conducted a *strengths, weaknesses, opportunities, and threats* framework analysis to explore the implied enablers of and barriers to the use of ICT interventions.

**Results:**

Of the 1149 identified articles, 31 (2.7%; n=4185 participants) met the inclusion criteria. Of the 31 articles, 5 (16%) mentioned the use of various eHealth interventions. A range of technologies was reported, including mobile health (8/31, 26%), telehealth (7/31, 23%), electronic health record (2/31, 6%), and mixed ICT interventions (14/31, 45%). Various chronic diseases affecting older adults were identified, including congestive heart failure (9/31, 29%), diabetes (7/31, 23%), chronic respiratory disease (6/31, 19%), and mental health disorders (8/31, 26%). ICT interventions were all designed to help people self-manage chronic diseases and demonstrated positive effects. However, patient-related and health care provider–related challenges, in integrating ICT interventions in routine practice, were identified. Barriers to using ICT interventions in older adults included knowledge gaps, a lack of willingness to adopt new skills, and reluctance to use technologies. Implementation challenges related to ICT interventions such as slow internet connectivity and lack of an appropriate reimbursement policy were reported. Advantages of using ICT interventions include their nonpharmacological nature, provision of health education, encouragement for continued physical activity, and maintenance of a healthy diet. Participants reported that the use of ICT was a fun and effective way of increasing their motivation and supporting self-management tasks. It gave them reassurance and peace of mind by promoting a sense of security and reducing anxiety.

**Conclusions:**

ICT interventions have the potential to support the care of older adults with chronic diseases. However, they have not been effectively integrated with routine health care. There is a need to improve awareness and education about ICT interventions among those who could benefit from them, including older adults, caregivers, and health care providers. More sustainable funding is required to promote the adoption of ICT interventions. We recommend involving clinicians and caregivers at the time of designing ICT interventions.

## Introduction

### Background

Chronic diseases represent a significant public health challenge worldwide and are the predominant cause of death among older adults [1]. Older adults are also vulnerable to occupational injuries arising from the effects of chemical, physical, and biological exposure in the workplace. In 2016, approximately 70% of deaths and 40% of disability-adjusted life years because of occupational injuries occurred in persons aged ≥55 years [2]. The burden of chronic diseases such as cardiovascular diseases (CVDs), diabetes, neurological disorders, and musculoskeletal disorders falls heavily on older adults [3]. The population aged ≥60 years is expected to increase to 2 billion by 2050 worldwide [4]. Consequently, the global burden of chronic diseases among older adults is anticipated to rise [5,6]. Given the increasing prevalence of aging and chronic diseases, it is essential to focus on health care innovation to improve personal health services such as self-management. Self-management is based on the concept that people can learn to manage their health using their skills and resources and thus become less dependent on external agents [7].

Information and communication technology (ICT) has been used in several settings to help individuals diagnose, treat, and manage chronic diseases better [8]. ICT interventions in health care, which we define herein as eHealth, have been shown to be cost-effective for monitoring and controlling congestive heart failure, stroke, chronic obstructive pulmonary disorder (COPD), diabetes, hypertension, asthma, dementia, and depression [9-13]. ICT interventions have also been used to support caregivers [14]. For example, mobile health (mHealth) has the potential to reduce the caregiver’s work burden by supporting the monitoring of medication use and providing significant interaction with older adults, thus minimizing the need for hospitalization [15]. Hence, ICT interventions may provide a solution to some of the challenges of aging and chronic diseases. However, there is conflicting evidence regarding the effectiveness of using ICT interventions among older adults with chronic diseases. Some positive outcomes have been identified for simple telephone interventions [16], which in some cases generated similar outcomes to more complex technologies [17-19]. As per suggestions made by other authors, there are opportunities to explore and compare perceptions among direct service providers, older adults living with chronic diseases, and caregivers about the challenges of various types of ICT interventions in both high- and low-income countries [20-22]. Therefore, there is a strong impetus for exploring the efficacy of ICT interventions and how this effectiveness differs in various settings.

The current high use of ICT among young people shows that ICT could be a future intervention model in health care, enhancing the number of people in need who are reached [23]. However, the approach of older adults to internet and health technology differs from that of younger people. Older adults may have lower rates of computer use and health-related internet use than younger adults [24]. Indeed, Heart et al [25] found that older adults require some skills to adopt the use of ICT interventions. Older adults with chronic diseases have also been reported to face numerous challenges such as altered cognition, visual and hearing difficulties, lack of trust, and privacy concerns as they encounter technology [26,27]. Without adopting these skills and addressing barriers, older adults might not receive the optimal benefits of ICT interventions in routine care. Hence, there is a critical need to better understand and map the barriers associated with the use of ICT interventions among older adults with chronic diseases to maximize the future uptake of ICT interventions and support personalized health care [28]. It is also essential to identify enablers of the use of ICT interventions so as to facilitate the design of mitigating strategies to overcome the barriers to use. Most ICT interventions described in the literature have targeted children, adolescents, or younger adults. We are not aware of any previous systematic or scoping review of the enablers of and barriers to the adoption of ICT interventions for supporting older adults with chronic diseases.

### Objective

In this review, we aim to identify (1) the available ICT interventions that have been used for managing older adults with chronic diseases and (2) the barriers to and enablers of using ICT interventions among older adults with chronic diseases.

## Methods

### Design

This scoping review was conducted using the PRISMA-ScR (Preferred Reporting Items for Systematic Reviews and Meta-Analysis Extension for Scoping Reviews) guidelines [29] and adopting the Arksey and O’Malley [30] framework. This framework outlines five stages for completing a scoping review: (1) identifying the research question; (2) identifying relevant published reports; (3) publication selection; (4) charting the data; and (5) collating, summarizing, and reporting the results [30], all of which have been followed in the conduct of this review.

### Database Selection and Search Strategy

A literature search was performed using 4 databases: Ovid MEDLINE, Embase, Scopus, and PsycINFO. We also used the ProQuest database to include eligible papers and proceedings published in association with computer science and technology conferences. We included articles and conference papers published from January 2000 to July 2020, which had full text in English and were peer reviewed. We selected the time frame of the past 2 decades to identify recent work undertaken on ICT interventions among older adults with chronic diseases. The population of older adults with chronic diseases could benefit from targeted health education interventions. We defined older adults as those ≥aged 55 years [31], so only studies with this definition were included. The search strategies were drafted through team discussions and checked and revised by an experienced librarian. We used the following search terms: *information and communication technology* or *mHealth* or *mobile health* or *telehealth* or *eHealth* or *remote monitoring* or *clinical decision support system* or *mobile phone technology* or *electronic health record* and *arthritis* or *asthma* or *back pain* or *carcinoma* or *cardiovascular disease* or *chronic obstructive pulmonary disease* or *diabetes* or *mental health* or *non-communicable diseases* or *chronic diseases* and *ageing* or *elderly* or *older adults* or *55+ age group* and *barriers* or *enablers* or *challenges* or *opportunities* or *benefits* or *threats*. We included eight major groups of chronic diseases in the review: arthritis, asthma, back pain, cancer, CVDs, COPD, diabetes, and mental health conditions. Multimedia Appendix 1 contains the search strategies and Boolean expressions for each database.

A total of 2 reviewers (SBZ and RKK) screened the titles and abstracts of the selected articles and identified duplicates. In cases of conflicting opinions regarding the eligibility of specific articles, the reviewers discussed their views with a third reviewer (SMSI) to reach a consensus. If inclusion was unclear from the title, the abstract was screened. Similarly, if inclusion was unclear from the abstract, the reviewer read the full text. We included original articles, all types of reviews, and conference papers (Table 1) for this scoping review. Once we identified suitable articles, we also looked for qualitative data included in the analysis. Here, we particularly looked for specific information related to barriers, enablers, and uses of ICT for supporting the care of older adults with chronic disease.

### ICT Types and End Users

Our definition of ICT interventions in health care, otherwise known as eHealth, includes the following: mHealth, electronic health records (EHRs), clinical decision support systems (CDSSs), telehealth and telemedicine, virtual reality in health care, and information technology systems used in health care settings. mHealth includes the use of mobile phones, mobile apps, PDAs, and PDA phones (eg, smartphones and handheld and ultraportable computers such as tablet devices) [11]. Telemedicine and telehealth are considered subdomains of eHealth and comprise communication networks to deliver health care interventions from one geographical location to another [32]. A remote monitoring system is defined as a subset of mHealth and telemedicine, which uses sensors to generate patient data.

We use the following ICT terminology in this paper:

- ICT device: refers to hardware only
- ICT intervention: refers to a specific program of research or implementation of ICT (eg, computer, mobile phone or tablet apps, and telehealth)

We considered older adults living with chronic diseases, their caregivers or family members, and health care providers as end users of ICT interventions.

### Data Extraction and Synthesis

SBZ, RK, and SMSI developed a data extraction form based on the aims of this review. SBZ and RK extracted data on the article title, names of first authors, publication year, study types or methods, setting, sample size, findings or recommendations, and expected or experienced barriers for all selected articles. Outcomes related to the use of ICT interventions were presented as *positive*, *no difference* or *negative* based on the conclusion reported in the included articles. No negative or neutral (no difference) outcomes were identified. In the case of qualitative data, factors related to barriers and enablers were coded in the data extraction form according to themes that emerged from the studies.

Second, we described and identified various ICT interventions—mHealth, EHR and CDSS, telemedicine, and remote monitoring—that were used for older adults with chronic diseases. Third, we reviewed articles to identify challenges in using ICT interventions among older adults with chronic diseases. For example, factors such as lack of motivation, comorbidities, poor adherence to treatment following ICT interventions, and absence of prior experience in the operation of ICT devices for older adults were considered as challenges. Issues related to costs of implementation, infrastructure, data security, and delays in making a decision were considered in the implementation category. Finally, we conducted a strengths, weaknesses, opportunities, and threats (SWOT) [33] analysis to explore the enablers of and barriers to the use of ICT interventions among older adults with chronic diseases. We used a codebook for the domains of *strength*, *weakness*, *opportunity,* and *threat* to report a descriptive analysis. Before this qualitative analysis, strategies for data coding were identified. SBZ and RK independently read and coded the articles. Each of the domains of SWOT was grouped into two categories: *patient-related factors (operational)* or *health care provider–related factors*. The patient-related category included factors associated with ICT interventions, which we define as *operational* here. We then applied this conceptual framework to identify emerging themes in each of these categories from the selected articles. Codes were then grouped into categories and eventually aggregated into 4 domains. After the initial round of coding, the 2 coders met with a senior researcher (SMSI) to cross-check the coding; thus, a final set of codes was agreed upon. The reviewers used Microsoft Excel 2014 to sort the articles.

## Results

### Overview

**Figure 1:**
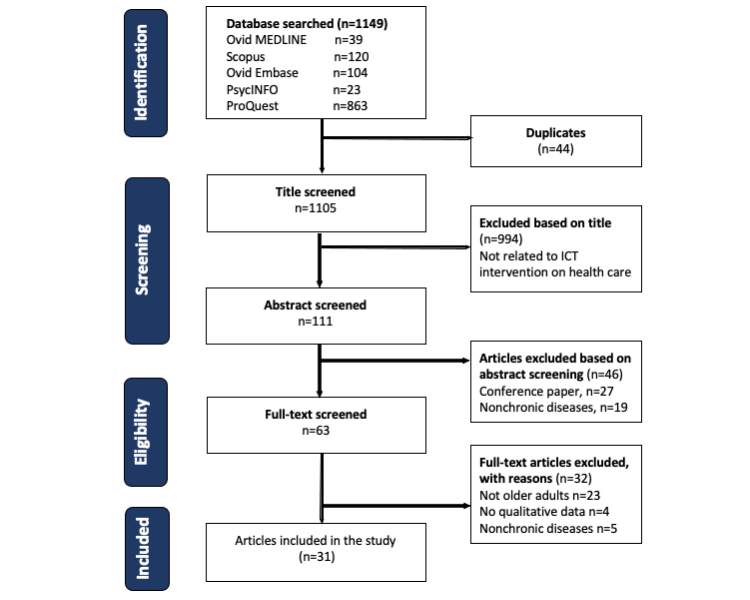
Flowchart of the literature search used for the selection of articles. This flowchart provides information regarding the various phases of the investigation, including the number of articles identified and the number included and excluded following the PRISMA-ScR (Preferred Reporting Items for Systematic Reviews and Meta-Analysis Extension for Scoping Reviews) guidelines. ICT: information and communication technology.

A total of 1149 articles, including conference papers (863/1149, 75.12%), were identified. Of the 1149 articles, 44 (3.83%) were duplicates (Figure 1). We excluded 86.51% (994/1149) of articles that were either not related to ICT interventions for older adults with chronic diseases or studies already reported in the systematic reviews that we included. Of the 1149 articles, after screening the titles and abstracts, 46 (4%) additional articles were excluded, leaving 63 (5.48%) articles for full-text screening. Of the 63 articles, there were 4 (6%) conference papers that were mostly based on formative research (design and development). As these papers lacked both quantitative and qualitative data (patient recruitment and barriers to and enablers of using ICT), we did not include them in the final selection. Finally, of the 63 articles, 26 (41%) were excluded following a full-text review, with 31 (49%) articles remaining (Figure 1).

### Characteristics of Articles Included in the Review

**Figure 2:**
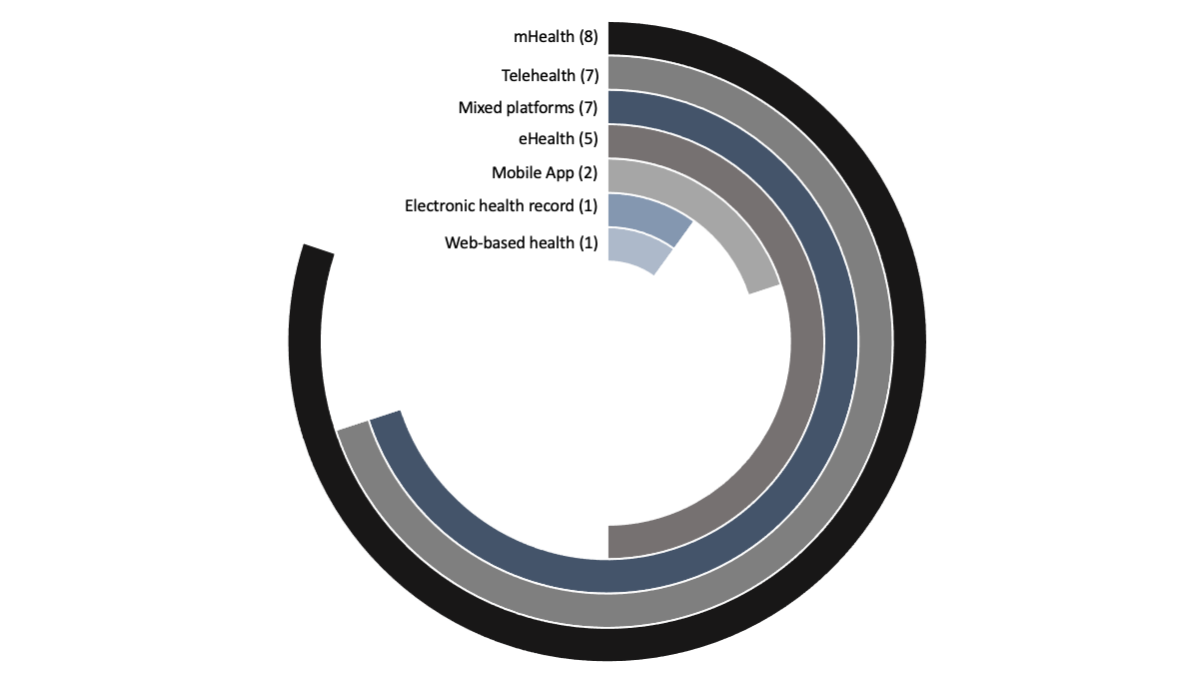
Multilayered donut chart shows the distribution of information and communication technology interventions used in health care. This figure shows various information and communication technology interventions that have been primarily described in the included studies or reviews in our scoping review. The total number of studies or reviews that mentioned various information and communication technology interventions is included in the bracket. mHealth: mobile health.

The characteristics of the included articles are presented in Table 1. Of the 31 included papers (total number of participants, n=4185), 2 (6%) were randomized controlled trials (RCTs) [34,35], 10 (32%) described non-RCT design intervention studies [36-45], and 13 (42%) were review articles [46-58]. These 13 review articles comprised 4 (31%) systematic reviews [46,48,53,56] and 2 (15%) scoping reviews [50,58]. In addition, 19% (6/31) were conference papers that described cross-sectional studies [59-64] (Figure 2).

**Table 1:** Characteristics of included articles.

Study	Country	Study design or type of article	ICT^a^ interventions	Instrument	Sample or articles	Target condition	Findings or recommendations	Limitations or challenges of ICT interventions
Miguel et al, 2013 [34]	Australia	RCT^b^ (6-month study period)	Telehealth intervention	Face-to-face interviews	80	COPD^c^	The telehealth group had comparatively fewer hospital admissions and a reduced length of stay than the control group.	Maintenance cost (high)
Barbera et al, 2018 [35]	Finland, France, and the Netherlands	RCT	Internet-based approaches	N/A^d^	2725	Dementia, CHF^e^, DM^f^, and dyslipidemia	Participants in the intervention arm were motivated to access information, advice, and motivational support throughout the intervention.	High cost and country-specific adaptation were major limitations
Barron et al, 2014 [36]	United States	Qualitative	Patient portal (EHR^g^)	Cognitive walkthrough	14	COPD and CHF	Patients with chronic diseases and caregivers were satisfied using the patient portal.	Assistance required for portal use Medical terms (unfamiliar)
Bhattarai et al, 2020 [37]	Australia	Qualitative	App for self-management of pain	Semistructured interviews	6	Arthritic pain	Apps for self-management of pain were potentially valuable for older patients App’s content and usability features should be relevant to the users	Apps were required to meet the user’s needs Pain self-management app might not be helpful if not designed to be used friendly
Chang et al, 2017 [38]	Taiwan	Qualitative	Telehealth	Semistructured (technology acceptance model)	18	DM	Participants with diabetes self-managed their disease with the help of telehealth	Mixed feelings regarding dependence on others for telehealth related problem solving
Coley et al, 2019 [39]	Finland, France, and the Netherlands	Mixed	eHealth intervention or internet counseling	Web-based questionnaire and semistructured interviews	343	CVDs^h^ and diabetes	Altruism and personal benefits were motivations for older adults’ use of telehealth Prevention of functional dependency on caregivers was a main underlying motivation	Internet-based health information perceived as unreliable by older adults Specific practical advice and encouragement was required for making lifestyle changes
Kim et al, 2019 [40]	United States	Mixed	Telehealth	Web-based surveys and in-depth interviews	20	Depression care	Telehealth was perceived as useful for managing symptoms and reducing costs.	Reimbursement and cost-related factors Patient home environment (not suitable) Agency-related characteristic (not well equipped)
Zettel-Watson et al, 2016 [41]	United States	Cross-sectional- exploratory study	Web-based health management tools	Web-based survey	169	Chronic diseases	Most users (89%) were satisfied with web-based health management tools Users were more likely to be younger, female, and married	Privacy or security was a concern among participants Users were not adequately aware of the exact benefits of web-based health management tools
Lee et al, 2016 [42]	United States	Pilot study	Android tablet with an installed app	A mobile-based health technology intervention	18	CVDs and CHF	Knowledge of self-management (anticoagulation) significantly improved from baseline to follow-up Participants were satisfied with the simplicity of the app	Some health care providers were not receptive to their patients using mHealth^i^ apps Privacy and security of information was a concerned
Mirza et al, 2008 [43]	New Zealand	Pilot study (qualitative nature)	mHealth initiative (through SMS text messaging)	Semistructured interviews	18	Diabetes and heart disease	High acceptability and recognition of the advantages of mHealth Issues affecting mHealth adoption, such as social issues, technical issues, economic issues, clinical or organizational issues	Patients’ access to their EHR was recommended by the health care providers Impaired abilities to cope with technology
Radhakrishnan et al, 2016 [44]	United States	Qualitative	Telehealth	Semistructured interviews	23	Cardiac disease, pulmonary disease, and DM	Positive impact on cost-effectiveness and patient-centered outcomes Home health management culture was important Establishment of patient–clinician and interprofessional communication was required	Factors negatively affected the telehealth program: Financial challenges Technical issues Management and communication-related issues
Nymberg et al, 2019 [45]	Sweden	Qualitative	eHealth (EMR^j^, telehealth, and mHealth)	Focus group interviews	15	Hypertension, diabetes, and COPD	Mixed feelings toward eHealth by the older adults Participants reported dissatisfaction in accessing health care	Lack of will, skills, self-trust, or mistrust in the new technology Organizational barriers (poor IT^k^ systems)
Rocha et al, 2019 [46]	N/A	Systematic review	mHealth	A systematic review of reviews and meta-analyses	66 reviews	DM, mental illness, cancer, COPD, and CVDs	mHealth interventions had positive effects on various health-related outcomes, including medication adherence No adverse impact of mHealth was identified	More research-based evidence was recommended for the incorporation of mHealth in clinical practices
Searcy et al, 2019 [47]	N/A	Narrative review	mHealth technologies	—^l^	—	CVDs	mHealth interventions for older adults with cardiovascular disease yielded mixed results	Physical limitations and cognitive challenges were identified as limitations
Peek ST et al, 2014 [48]	N/A	Systematic review	Electronic technologies	—	16 articles	Chronic diseases	Apparent benefits of using mHealth were recommended for widespread acceptance	Lack of security in using mHealth was a concern
Vollenbroek-Hutten et al, 2017 [49]	N/A	Narrative review	Various ICT platforms	—	673	Chronic pain, COPD	Patients were satisfied with ICT-supported services	Real-time contact and safe monitoring of patients in an emergency was challenging
Wildenbos et al, 2018 [50]	N/A	Scoping review	mHealth	Framework analysis	—	Chronic diseases	A total of 4 critical categories of aging barriers influencing usability of mHealth were cognition, motivation, physical ability, and perception	Obstacles related to cognitive and physical ability to use mHealth was difficult for older adults to overcome
Blass et al, 2006 [51]	United States	Narrative	Telehealth	Ethics and public policy (ethical challenges)	—	Physical or psychiatric illness	Ethical challenges with homebound older patients were unique because of patient characteristics and features of the treatment environment.	Protecting the confidentiality of personal information of users could be challenging
Bostrom et al, 2020 [52]	N/A	Narrative review	Various mHealth technology	mHealth cardiac rehabilitation	—	CVD, hypertension, arrhythmia, and CHF	mHealth: cardiac rehabilitation represented a particularly attractive area compared with traditional barriers to facility-based cardiac rehabilitation Improved accessibility to patients unable to attend traditional cardiac rehabilitation	Safety of mHealth-based cardiac rehabilitation Physical limitations (eyesight and fine motor skills) might limit use in older adults Hesitance from older adults to adopt technology
Christensen et al, 2020 [53]	N/A	Systematic review	Video consultations	Different survey instruments	21 studies	Mental health practice (unipolar depression)	Video consultations were found to be a viable option for delivering mental health care Video consultations allowed patients to receive treatment at their home	Incorrect diagnosis Required trained health care providers
Gilbert et al, 2015 [54]	United States	Narrative	Gerontechnology: mHealth	Applications of gerontechnology by stakeholders	—	Chronic diseases	A digital divide was developed between older adults and younger adults Gerontechnology was found to be an essential limb of mHealth unique to older adults	Without focusing on user-centered design, it would be difficult to widen the accessibility and engagement of older adults in the long run
Henriquez-Camacho et al, 2014 [55]	N/A	Narrative review	eHealth technologies	Problems related to age and technology	—	Chronic diseases	eHealth technologies were found to have the potential to improve access to health care by empowering patients	Difficulty in accessing eHealth care because of limited resources, lack of literacy, large geographical areas, and physical, cognitive, and visual impairment
Harerimana et al, 2019 [56]	N/A	Systematic review	Telehealth interventions	Users’ perceptions of a telehealth intervention	13 articles	Chronic diseases	Use of telehealth reduced emergency visits, hospital admissions, and depressive symptoms and improved cognitive functioning of the patients	Obstacles for using telehealth were levels of education, cognitive function, living arrangement, and negative experience with the clinics
Jimison et al, 2008 [57]	N/A	Narrative review	Health IT	Barriers and drivers to the use of health IT	129 articles	Chronic diseases	Rapid and frequent interactions from a clinician improved use and user satisfaction	It was critical that data entry does not feel cumbersome and that the intervention fit into the user’s daily routine.
Matthew-Maich et al, 2016 [58]	N/A	Scoping review	mHealth	Designing, implementing, and evaluating mHealth technologies	42 articles	Chronic diseases	The implementation of mHealth technologies in home-based care for older adults and self-management of chronic conditions are important areas for further research.	A user-centered and interdisciplinary approach is imperative to enhance the feasibility and acceptability of mHealth innovations
D’Haeseleer et al, 2019 [59]	Italy	Conference paper	Various ICT platforms for self-monitoring services	Focus group interview	12	Chronic diseases	The skills to use computers were heterogeneous among the older adults They perceived the use of health technologies as a threat to social interaction	Health technologies are not ready for adoption by older adults yet, and further research on making them more accessible is required
Hosseinpour et al, 2019 [60]	Iran	Conference paper	Telecare	Medical records	38	Acute coronary syndrome	An innovative telecare system based on artificial intelligence is presented for the early diagnosis of acute cardiac syndrome	Improving the accuracy of the telecare system by using real-time information of users was challenging
Lorenz et al, 2007 [61]	Germany	Conference paper	mHealth	Semistructured interviews	8	Chronic diseases	Older adults prefer the advanced interface, characterized by displays of graphical symbols and animations, of devices They also preferred the basic interface with simple navigation over 2 different screens	Participants preferred a device like the shape of a wristwatch, equipped with an unobtrusive system It was challenging to develop a tool for all such older versus younger patients and persons with computer experience versus no computer experience instances
Pikna et al, 2018 [62]	Slovakia	Conference paper	ICT	Semistructured interviews	5	Chronic diseases	Older adults usually use a mobile phone or a computer to share their experiences with others on different social networks	The use of ICT can be a difficult challenge for seniors.
Termeh et al, 2015 [63]	Iran	Conference paper	Smart-watches and sensors	Implementation of a U-Health^m^ system	—	Heart failure and arterial fibrillation	U-Health approach is relatively low cost, can be implemented using simple equipment, and does not limit the movement of the patient.	To get the notification patient has to have the watch on his or her wrist.
Wang et al, 2018 [64]	United States	Conference paper	ICT	Semistructured interviews	12	Chronic diseases	Older adults were positively influenced for using new technologies	Difficulty in accessing ICT care due to limited resources and lack of literacy

^a^ICT: information and communication technology.

^b^RCT: randomized controlled trial.

^c^COPD: chronic obstructive pulmonary disorder.

^d^N/A: not applicable.

^e^CHF: chronic heart failure.

^f^DM: diabetes mellitus.

^g^EHR: electronic health record.

^h^CVD: cardiovascular disease.

^i^mHealth: mobile health.

^j^EMR: electronic medical record.

^k^IT: information technology.

^l^Not available.

^m^U-Health: ubiquitous health.

In total, the systematic reviews used in the current synthesis included 122 independent studies. We did not include studies already reported in the systematic reviews as individual studies to avoid duplication. Clinical trial intervention studies (RCTs and non-RCTs) were conducted in Finland, France, the Netherlands [35,39], Taiwan [38], the United States [36,40-42,44,64], Australia [34,37], New Zealand [43], Germany [61], Slovakia [62], Italy [59], and Sweden [45]. Except for Iran [60,63], no studies were conducted in low- to middle-income countries (LMICs). Most of the studies, except 1 [41], were pilot studies or short-term interventions. Original articles were either qualitative [36-38] or used mixed methods [39,40]. Various methods were used to measure the outcome of interest, including cognitive walk-throughs [44], semistructured interviews [37,39,42-44,61,62,64], in-depth interviews [40], focus groups [45,59], and web-based surveys [39-41]. The Technology Acceptance Model [38] and the Unified Theory of Acceptance and Use of Technology Model [48] were also used to assess the feasibility of ICT interventions in 2 studies.

### ICT Interventions Used in Health Care

All articles provided evidence that ICT interventions are beneficial for health care among older adults with chronic diseases (Table 1). We identified various ICT platforms used for supporting health care providers as they manage chronic diseases in older adults. A total of 3 studies and 2 reviews mentioned the use of ≥1 mixed eHealth intervention such as electronic technologies, internet counseling, video consultation, EHR, and telehealth [39,45,46,48,55]. A total of 3 studies and 5 reviews, including 2 scoping reviews, focused particularly on mHealth [43,46,47,50,52,54,58], including mobile apps [37,42]. A total of 4 studies and 2 reviews focused on telehealth [34,38,40,44,51,56]. One study specifically focused on the use of a patient portal or EHR [36]. One study was on a web-based health management tool [40] for chronic care. Finally, 7 further reviews incorporated the use of a combination of ICT interventions [49,53,57], including EHR, mHealth, and video consultation, in providing care for older adults with chronic diseases. Figure 2 shows the distribution of ICT interventions that have been primarily used or described in the included original articles or reviews.

All the included articles reported a positive outcome for supporting the management of chronic diseases such as CVDs (eg, chronic heart failure, atrial fibrillation, and hypertension) [36,39,42-47,52,57], diabetes [35,38,39,43-46], COPD [34,36,44-46,49], dyslipidemia [35], arthritic pain [37,49], mental illness including depression and dementia [35,40,46,51,53], and cancer [46]. Thus, there were no reports of neutral or negative effects that might underdetermine the use of ICT interventions.

### Challenges to and Enablers of Implementing ICT Interventions in Health Care

Multimedia Appendix 2 [34-54,56-64] describes the primary SWOT assessment outcomes.

#### Strengths

##### Patient-Related Factors

In many cases, identified in 48% (15/31) of articles, participants reported that the use of an ICT intervention was a fun or effective way for improving health [37,39,43,46-48,52-56,59,61,62,64] by increasing their motivation and supporting self-management tasks [38,42-45,47,50-52,54-57,59,61-63]. Approximately 48% (15/31) of articles identified that patients were frequently satisfied with using 1 or a combination of ICT interventions [34,41-44,46-49,52,55-57,62-64]. They encountered fewer face-to-face interactions with clinical staff and with other patients [34,35,37,39,41-45,48,52-55,57-62,64], thus mitigating their functional dependency [34,35,38,39,43,44,46-48,50,51,54,55,57-59,61-63] on clinical or hospital services. The use of ICT interventions gave them reassurance and peace of mind [34,35,43-45,47,48,50-52,57-59,61-64] by improving a sense of security and reducing anxiety [34,43,44,47,48,51,52,54,57,59-64]. Older adults with chronic diseases who participated in studies reported getting direct access to treatment and benefited from additional medical monitoring when they felt unwell. The use of ICT interventions also encouraged them to continue physical activity, maintain a healthy diet, and stop smoking [37,43-47,49,52,57,58,60-62,64].

##### Health Care Provider–Related Factors

One of the biggest advantages of ICT interventions that was identified was their nonpharmacological nature [35,39,42-46,48,51,52,54-57,61-64]. This point was made in 58% (18/31) of articles, with a particular focus on the value, for managing older adults with chronic disease, of providing health education and regular follow up. Health care providers reported the use of interactive push-notification features [38,43,46-50,54-57,59,60,62], larger screens [34,36,48,52,57,61] and written instructions [36,48,50] for ICT devices as helpful. Health care providers also expressed a desire to get more available functions, such as voice demonstration and video chatting, for integrating ICT interventions into routine systems (mentioned in 9/31, 29% articles) [41,46-48,50,52,54,55,61].

#### Weaknesses

##### Patient-Related Factors

The most common limiting factor, identified in 35% (11/31) of articles, was the lack of confidence in computer skills [40,45,47,48,50,54-56,59,61,64]. In addition, inconvenience arising from the need to have a continuous internet connection was identified in 48% (15/31) of articles [35,39,43-45,48,50,53-57,62-64]. Approximately 39% (9/23) of articles identified that participants felt embarrassed when they failed to correctly operate ICT devices [38,43-45,48,50,53,55-57,59,61,62,64]. As a result, they were sometimes dependent on other family members to operate the devices. This dependency made some people feel uncomfortable and concerned about bothering their family members for assistance with ICT devices [37,38,48,50-52,54,55,59]. Approximately 32% (10/31) of articles identified instances when participants did not voluntarily learn to use the ICT devices if their family members could operate it for them [38,42,47,48,50,52,54-56,61]. Participants also required support (supervision) for adhering to disease management behaviors [34,38,42,48,50,53,58,59,61-64] and maintaining their ICT devices. Some people were concerned regarding the potential loss of data or lack of protection of their privacy [41,44,45,48,51,52,54,55,61] when using ICT interventions. Approximately 39% (12/31) of articles identified that older adults lacked confidence in the use of an internet-based intervention, even if they had the necessary computer skills [40,42,48-50,53-56,59,61,64]. Some participants reported inconveniences associated with the ICT device itself, such as small screens or cramped keyboards [43,46-49,52-55,62] or inadequate battery life lasting 4 to 5 hours [43,46-48,58,61-64]. Approximately 39% (12/31) of articles reported that participants found the ICT devices hard to use because of a lack of familiarity with the medical terms used in the instructions of these devices [36,41,48,52-55,57,59,61,62,64].

##### Health Care Provider–Related Factors

Only a few weaknesses were reported for health care provider–related factors. Health care providers reported that some older adults with chronic diseases were dependent on family members or friends for using their ICT devices [38,47,50,52,61]. Hence, these participants, who were dependent on others, were sometimes not interested in learning how to operate the technology independently. In such cases, health care providers sometimes found it difficult to directly interact with patients using ICT interventions. An additional list of barriers to and challenges for the use of ICT interventions synthesized from current evidence is provided in Multimedia Appendix 3.

#### Opportunities

##### Patient-Related Factors

The authors of 58% (18/31) of articles reported that ICT interventions supported older adults in maintaining regular medical checkups [34,35,39,43-46,48,50,55-59,61-64] and attaining benefits from lifestyle changes [34,35,39,43-45,48,50,52,53,55-60,62-64]. The authors (19/31, 61% articles) also reported that most participants received encouragement from physicians and nurses to use ICT interventions [34,35,39,43-45,48,50,52,53,55-59,61-64] and develop their self-care disease management skills [34,40-42,45-50,53-55,58-64]. Most participants were partially willing to pay for taking up the ICT interventions [48,50,53-61,63,64] if they were affordable. Most of the participants, identified in 35% (11/31) of articles, were also keen to recommend the ICT interventions to others [43,44,48,50,52,54,55,57,59,61,62].

A range of operational factors was identified in relation to the use of hardware and software related to ICT interventions. Most of the investigators reported that the local context should be considered during the development of an ICT intervention [34,37,44,48,52-55,57,59-61]. For example, a mobile app should have personalization features to suit the user’s preferences in their language [34,37,46-50,55,56,59-62,64]. Furthermore, participants wanted the ICT devices to be portable, rechargeable [38,43,46-49,53,56,59-63], simple, and easy to use [38,43-49,52-58,61-63].

##### Health Care Provider–Related Factors

In 48% (15/31) of articles, providers reported that they were satisfied that the ICT interventions allowed them to give special care to older adults with cognitive or sensory dysfunction [38,43,45-47,49,52-56,58,59,61,62,64]. There was consensus that clinicians’ active involvement is crucial for the integration of an ICT intervention into a self-management strategy [34,37,41-45,50-52,55-58,61-63].

#### Threats

##### Patient-Related Factors

The authors of 32% (10/31) of articles reported that some older adults had hearing and sight impairment and that these disabilities restricted communication with health care providers [35,38,46,50,52,53,56,62]. Cost was another factor, which was identified in 39% (12/31) of articles, that influenced the uptake of ICT interventions. Despite significant improvement in the self-care ability of patients, participants were unwilling to continue ICT interventions that attracted a fee [36,38,43,44,50,53-57,61,62]. For example, a home telehealth program could not be sustained because of financial challenges, technical complexities, and communication-related issues, even after operating for 12 years [44]. When the participants perceived a new ICT intervention as expensive and complex [38,45,46,50,53-55,58,59,62], they lost interest in using that intervention [38,47,48,50,52,59]. Some participants reported that a breach of confidentiality [37,51,53,56] occurred while using an ICT intervention.

##### Health Care Provider–Related Factors

The authors of 23% (7/31) of the articles reported that providers were influential in motivating their patients to use or stop the use of ICT interventions [41,43,44,50,53,55-57,59,62]. For example, patients were found to stop using an ICT intervention if their physicians did not encourage them to use the respective intervention [41,43,53-55,57-59,61,62,64]. Most health care providers believed that ICT interventions should only be deemed as an adjunct to the medical management of chronic diseases. However, some providers expressed concerns regarding the widespread use of ICT interventions replacing traditional health care delivery models (mentioned in 10/31, 32% articles), which could result in job loss [35,40,45,51,54,55,58,60,61,63].

## Discussion

### Principal Findings

Overall, findings from this scoping review highlight the potential benefit of ICT interventions or eHealth (eg, mHealth and mobile apps, EHR, remote monitoring, CDSS, and telemedicine) for supporting older adults in self-managing chronic diseases. The review highlighted a range of operational and technical barriers to using these ICT interventions for older adults. Our review highlighted age-related barriers to using ICT interventions, including cognition, motivation, physical limitations (eyesight and fine motor skills), and perception, which limited the use of ICT interventions among older adults with chronic diseases. In this case, personalized learning may meet the unique needs, interests, and capacities of individual users to mitigate these limitations [65]. Some of these limitations could be resolved via design optimization of ICT interventions, such as increasing the screen contrast to mitigate the loss of visual acuity or simplifying task movements to facilitate ICT use in patients with arthritis or physical disability [41]. A number of challenges and enablers in integrating ICT interventions into routine practice were also identified. Most of the included studies were pilot or short-term interventions conducted in a controlled environment. Hence, longitudinal studies aimed at assessing the long-term effectiveness of ICT interventions should be a priority.

Our results indicate that some older adults with chronic diseases might have reservations when it comes to engaging with ICT interventions. We found operational and technical challenges, including a lack of willingness to adopt new skills, poor confidence, and the lack of necessary skills to operate ICT devices. These findings are consistent with the results of other studies where older people expressed no interest in using novel technology and struggled to think of the need for such an application in their own lives [66,67]. Acceptance of these electronic or digital technologies may be more difficult for the current generation of older adults who did not grow up with these technologies [68,69]. Mitigating strategies to increase ICT literacy using short e-learning courses (eg, 2 weeks with 10-minute sessions each day) have been shown to be suitable for older adults [70,71].

We found strong motivation and desire to use ICT interventions among older adults with chronic diseases because of the nonpharmacological nature of the intervention. Self-management of chronic diseases includes the maintenance of a healthy lifestyle and adherence to medication. However, older adults seem to require specific motivation to make practical changes, such as eating a healthy diet and being physically active, even if they are already aware of their value [36]. Nilsen et al [72] reported that the traditional approach of episodic care provided in the clinic or through hospital support systems might not be sufficient to prevent chronic diseases without incorporating ICT interventions in health care. Therefore, health care providers are motivated to use ICT interventions to communicate with their patients to know whether they follow their advice.

It is imperative to understand the duration that people require to achieve a cost-effective outcome from ICT interventions. Findings from this scoping review suggest that older adults living with chronic diseases and caregivers were unwilling to pay for the use of ICT interventions, although they were happy with the service. Most participants only offered to pay partially. An explanation for this result is that all participants in the studies we reviewed were from high-income countries and frequently reported the lack of an appropriate insurance scheme and reimbursement for procuring devices required for ICT interventions. Without addressing the payment model, it will be challenging to ensure the proper use of ICT interventions in health care, even if older adults desire to use them. Chen and Chan [73] also reported that implementation costs were not adequately highlighted in designing specific ICT interventions in many countries. Therefore, the high cost seems to be a critical factor in determining the ability of an older adult to accept these interventions. Similarly, we also reported a home telehealth program’s failure after more than a decade of use because of financial challenges [44]. Hence, more sustainable funding and reimbursement are essential for promoting the adoption of ICT interventions.

In addition to the financial factors discussed above, this review highlights workload as an additional determinant of the adoption of ICT interventions. Managing life-threatening events, such as arrhythmia or heart attack, requires an immediate response from health care providers, and such a rapid response can be challenging to execute in many places, particularly in hard-to-reach areas. Failure to react to patients immediately may exacerbate the health risks of older adults with chronic diseases and render health care providers susceptible to accusations of negligence [74]. There is also the risk of generating false-positive alarms from these ICT interventions, which may require physical verification. Thus, such alarms could increase the workload of clinicians if they are required to personally evaluate every call. This may partly explain why not all clinicians were receptive to their patients using ICT interventions. Training can be a significant factor that influences health professionals’ eagerness to use or refer their patients for using ICT interventions at home [75-77].

Future app developers should consider involving end users in the design and development process for ICT interventions. We reported that clinicians’ involvement in the recruitment process appeared to influence the decision of participants to take part in the trials or studies. Hence, their involvement will be crucial for motivating patients to use ICT interventions. The authors also point out the necessity of ensuring that health care providers are encouraged and committed to recommending ICT interventions for their patients [13,78]. Otherwise, the willingness to use ICT interventions will never develop among patients, despite their ability to operate these devices. The general assumption that education is a relevant factor in adopting the use of ICT may not always be accurate, with the authors of an article reporting that level of education was not positively associated with the uptake of ICT interventions in the sample of patients they studied [79]. Health care providers can be an additional barrier to the adoption of ICT interventions by older patients. For example, Smelcer et al [80] reported that 30% of EHR system implementations worldwide failed because of their underutilization or inappropriate use by the clinician. They identify the concept of *medical authority*, where clinicians or health care providers affect medical practices such as diagnosis and management of chronic diseases for their patients, as critical for the implementation of EHR [81]. It seems likely that medical authority is also an essential factor in the implementation of other forms of ICT interventions.

Management of chronic diseases may require the engagement of multiple health care service providers [82]. This arrangement could be too complex for older adults with chronic diseases who are disabled or living in rural areas, particularly in hard-to-reach areas. Here, ICT interventions can play a significant role by offering interconnectedness among multiple providers. For example, some ICT interventions (CDSS and EHR) provide valuable features such as sharing data with other providers (interoperability) and providing patient-specific information such as drug adherence [83]. In doing so, we also report that some participants raised ethical and legal concerns related to sharing data (eg, privacy and security) with several providers. These barriers can be overcome if clinicians, health care workers, and service providers are obliged to maintain confidentiality and report all harmful events associated with the use of ICT interventions [10,13,78,84].

There are opportunities for implementing ICT interventions in LMICs to support the care of older adults with chronic diseases. Approximately 6.5 billion people reside in LMICs, and the proportion of older adults within this population will increase in the near future in these countries [85]. Most intervention studies that we included were from high-income countries. However, very few were from LMICs. Most developing countries lack the necessary financial strength to fund and implement ICT interventions properly. The good news is that the governments of many LMICs are also interested in investing in deploying eHealth to enhance health services, particularly in remote areas [86]. Finally, ICT interventions should help patients self-manage chronic diseases with minimal support from health care providers or clinics. Hence, clinicians and health care providers are required to convince patients to use ICT interventions in addition to routine clinic visits. None of the included reviews on ICT interventions reported harm. However, there are challenges to the implementation of these ICT interventions, particularly for older adults with chronic diseases. The provision of ICT literacy by health care providers and user-centered design by app developers may help older adults widen their engagement with ICT interventions [54]. Hence, longitudinal studies aimed at assessing the long-term effectiveness of ICT interventions should be a priority. Another priority should be to determine whether ICT interventions are clinically effective and cost-effective when used by rural health care providers. Hence, we recommend conducting a systematic review of existing studies on ICT interventions to evaluate their efficacy.

### Limitations

To the best of our knowledge, this is the first review of its type to use the SWOT framework to identify strengths, weaknesses, opportunities, and threats for the use of ICT interventions to support the care of older adults with chronic diseases. A potential limitation of our approach is that we did not consult external experts during the review process. Nevertheless, by conducting a scoping review on this topic, we have defined the nature, extent, and range of research activities on ICT interventions for older adults with chronic diseases. Although we searched the literature exhaustively using 4 academic databases, in addition to ProQuest, there is a possibility that we missed some important studies. In this scoping review, we focused on providing an overview of the available research evidence on the use of ICT interventions in older adults with chronic diseases. Therefore, we included a good range of original studies, systematic reviews, and conference papers to help answer our research question. Importantly, none of the studies included in these reviews overlapped. We did not perform a critical appraisal of the literature, which was beyond the scope of our objectives (PRISMA-ScR checklist is given in Multimedia Appendix 4 [29]).

### Conclusions

ICT interventions might help support the care of older adults with chronic diseases by increasing adherence to treatment and healthy lifestyles. However, the incorporation of ICT interventions into medical practice is still challenging. The involvement of clinicians is crucial for motivating people with chronic diseases to adopt ICT interventions to support self-management. There is a need to improve awareness and training in the available and effective ICT interventions among older adults and health care providers. Widespread implementation of ICT interventions will also require more sustainable approaches to funding and reimbursement. We recommend involving clinicians and caregivers when designing ICT interventions and integrating them into routine medical care.

## Appendix

Multimedia Appendix 1 Search strategies for Ovid MEDLINE, Embase, Scopus, and PsycInfo databases.

Multimedia Appendix 2 Framework analysis of strengths, weaknesses, opportunities, and threats on the use of information and communication technology in health care among older adults.

Multimedia Appendix 3 List of barriers to and challenges for the use of information and communication technology.

Multimedia Appendix 4 PRISMA-ScR (Preferred Reporting Items for Systematic Reviews and Meta-Analyses Extension for Scoping Reviews) checklist.

## Abbreviations

CDSS: clinical decision support system
COPD: chronic obstructive pulmonary disorder
CVD: cardiovascular disease
EHR: electronic health record
ICT: information and communication technology
LMIC: low- to middle-income country
mHealth: mobile health
PRISMA-ScR: Preferred Reporting Items for Systematic Reviews and Meta-Analysis
RCT: randomized controlled trial
SWOT: strengths, weaknesses, opportunities, and threats

## References

[1] Ageing and life-course: world report on ageing and health 2015. World Health Organization.

[2] GBD 2016 Occupational Risk Factors Collaborators (2020). Global and regional burden of disease and injury in 2016 arising from occupational exposures: a systematic analysis for the Global Burden of Disease Study 2016. Occup Environ Med.

[3] Prince MJ, Wu F, Guo Y, Gutierrez Robledo LM, O'Donnell M, Sullivan R, Yusuf S (2015). The burden of disease in older people and implications for health policy and practice. Lancet.

[4] Ageing and health. World Health Organization.

[5] (2010). Global status report on noncommunicable diseases. World Health Organization.

[6] Gong JB, Yu XW, Yi XR, Wang CH, Tuo XP (2018). Epidemiology of chronic noncommunicable diseases and evaluation of life quality in elderly. Aging Med (Milton).

[7] Bodenheimer T, Lorig K, Holman H, Grumbach K (2002). Patient self-management of chronic disease in primary care. JAMA.

[8] Zaman SB, Hossain N, Ahammed S, Ahmed Z (2017). Contexts and opportunities of e-Health technology in medical care. J Med Res Innov.

[9] Jaana M, Sherrard H (2019). Rural-urban comparison of telehome monitoring for patients with chronic heart failure. Telemed J E Health.

[10] Sticherling C, Kühne M, Schaer B, Altmann D, Osswald S (2009). Remote monitoring of cardiovascular implantable electronic devices: prerequisite or luxury?. Swiss Med Wkly.

[11] Free C, Phillips G, Galli L, Watson L, Felix L, Edwards P, Patel V, Haines A (2013). The effectiveness of mobile-health technology-based health behaviour change or disease management interventions for health care consumers: a systematic review. PLoS Med.

[12] Finkel S, Czaja SJ, Schulz R, Martinovich Z, Harris C, Pezzuto D (2007). E-care: a telecommunications technology intervention for family caregivers of dementia patients. Am J Geriatr Psychiatry.

[13] Kobza R, Erne P (2007). End-of-life decisions in ICD patients with malignant tumors. Pacing Clin Electrophysiol.

[14] E-health and m-health applications for older people. Health Literacy Centre.

[15] Using mobile technologies for healthier aging. mHealth Alliance, United Nations Foundation.

[16] GESICA Investigators (2005). Randomised trial of telephone intervention in chronic heart failure: DIAL trial. BMJ.

[17] Cleland J, Louis A, Rigby A, Janssens U, Balk A, TEN-HMS Investigators (2005). Noninvasive home telemonitoring for patients with heart failure at high risk of recurrent admission and death: the Trans-European Network-Home-Care Management System (TEN-HMS) study. J Am Coll Cardiol.

[18] Jerant AF, Azari R, Martinez C, Nesbitt TS (2003). A randomized trial of telenursing to reduce hospitalization for heart failure: patient-centered outcomes and nursing indicators. Home Health Care Serv Q.

[19] García-Lizana F, Sarría-Santamera A (2007). New technologies for chronic disease management and control: a systematic review. J Telemed Telecare.

[20] Kruse C, Fohn J, Wilson N, Nunez Patlan E, Zipp S, Mileski M (2020). Utilization barriers and medical outcomes commensurate with the use of telehealth among older adults: systematic review. JMIR Med Inform.

[21] Kruse CS, Fohn J, Umunnakwe G, Patel K, Patel S (2020). Evaluating the facilitators, barriers, and medical outcomes commensurate with the use of assistive technology to support people with dementia: a systematic review literature. Healthcare (Basel).

[22] Kruse CS, Mileski M, Moreno J (2017). Mobile health solutions for the aging population: a systematic narrative analysis. J Telemed Telecare.

[23] Selwyn N (2003). ICT for all? Access and use of Public ICT Sites in the UK. Inf Commun Soc.

[24] Fischer SH, David D, Crotty BH, Dierks M, Safran C (2014). Acceptance and use of health information technology by community-dwelling elders. Int J Med Inform.

[25] Heart T, Kalderon E (2013). Older adults: are they ready to adopt health-related ICT?. Int J Med Inform.

[26] Xie B (2003). Older adults, computers, and the internet: future directions. Gerontechnol.

[27] Holzinger A, Searle G, Nischelwitzer A (2007). On some aspects of improving mobile applications for the elderly. Universal Acess in Human Computer Interaction.

[28] Hoque R, Sorwar G (2017). Understanding factors influencing the adoption of mHealth by the elderly: an extension of the UTAUT model. Int J Med Inform.

[29] Tricco AC, Lillie E, Zarin W, O'Brien KK, Colquhoun H, Levac D, Moher D, Peters MD, Horsley T, Weeks L, Hempel S, Akl EA, Chang C, McGowan J, Stewart L, Hartling L, Aldcroft A, Wilson MG, Garritty C, Lewin S, Godfrey CM, Macdonald MT, Langlois EV, Soares-Weiser K, Moriarty J, Clifford T, Tunçalp Ö, Straus SE (2018). PRISMA Extension for Scoping Reviews (PRISMA-ScR): checklist and explanation. Ann Intern Med.

[30] Arksey H, O'Malley L (2005). Scoping studies: towards a methodological framework. Int J Soc Res Methodol.

[31] (2001). Definition of an older person. Proposed working definition of an older person in Africa for the MDS Project. World Health Organization.

[32] Sood S, Mbarika V, Jugoo S, Dookhy R, Doarn CR, Prakash N, Merrell RC (2007). What is telemedicine? A collection of 104 peer-reviewed perspectives and theoretical underpinnings. Telemed J E Health.

[33] Madsen D (2016). SWOT analysis: a management fashion perspective. Int J Bus Res.

[34] De San Miguel K, Smith J, Lewin G (2013). Telehealth remote monitoring for community-dwelling older adults with chronic obstructive pulmonary disease. Telemed J E Health.

[35] Barbera M, Mangialasche F, Jongstra S, Guillemont J, Ngandu T, Beishuizen C, Coley N, Brayne C, Andrieu S, Richard E, Soininen H, Kivipelto M, HATICE study group (2018). Designing an internet-based multidomain intervention for the prevention of cardiovascular disease and cognitive impairment in older adults: the HATICE Trial. J Alzheimers Dis.

[36] Barron J, Bedra M, Wood J, Finkelstein J (2014). Exploring three perspectives on feasibility of a patient portal for older adults. Stud Health Technol Inform.

[37] Bhattarai P, Newton-John TR, Phillips JL (2020). Apps for pain self-management of older people's arthritic pain, one size doesn't fit all: a qualitative study. Arch Gerontol Geriatr.

[38] Chang C, Lee T, Mills ME (2017). Experience of home telehealth technology in older patients with diabetes. Comput Inform Nurs.

[39] Coley N, Rosenberg A, van Middelaar T, Soulier A, Barbera M, Guillemont J, Steensma J, Igier V, Eskelinen M, Soininen H, Moll van Charante E, Richard E, Kivipelto M, Andrieu S, MIND-AD, HATICE groups (2019). Older adults' reasons for participating in an eHealth prevention trial: a cross-country, mixed-methods comparison. J Am Med Dir Assoc.

[40] Kim E, Gellis ZD, Bradway C, Kenaley B (2019). Key determinants to using telehealth technology to serve medically ill and depressed homebound older adults. J Gerontol Soc Work.

[41] Zettel-Watson L, Tsukerman D (2016). Adoption of online health management tools among healthy older adults: an exploratory study. Health Informatics J.

[42] Lee J, Evangelista LS, Moore AA, Juth V, Guo Y, Gago-Masague S, Lem CG, Nguyen M, Khatibi P, Baje M, Amin AN (2016). Feasibility study of a mobile health intervention for older adults on oral anticoagulation therapy. Gerontol Geriatr Med.

[43] Mirza F, Norris T, Stockdale R (2008). Mobile technologies and the holistic management of chronic diseases. Health Informatics J.

[44] Radhakrishnan K, Xie B, Jacelon CS (2016). Unsustainable home telehealth: a Texas qualitative study. Gerontologist.

[45] Nymberg VM, Bolmsjö BB, Wolff M, Calling S, Gerward S, Sandberg M (2019). 'Having to learn this so late in our lives…' Swedish elderly patients' beliefs, experiences, attitudes and expectations of e-health in primary health care. Scand J Prim Health Care.

[46] Rocha N, Santos M, Cerqueira M, Queirós A (2019). Mobile health to support ageing in place: a systematic review of reviews and meta-analyses. Int J E Health Med Commun.

[47] Searcy RP, Summapund J, Estrin D, Pollak JP, Schoenthaler A, Troxel AB, Dodson JA (2019). Mobile health technologies for older adults with cardiovascular disease: current evidence and future directions. Curr Geri Rep.

[48] Peek ST, Wouters EJ, van Hoof J, Luijkx KG, Boeije HR, Vrijhoef HJ (2014). Factors influencing acceptance of technology for aging in place: a systematic review. Int J Med Inform.

[49] Vollenbroek-Hutten M, Jansen-Kosterink S, Tabak M, Feletti LC, Zia G, N'dja A, Hermens H, SPRINTT Consortium (2017). Possibilities of ICT-supported services in the clinical management of older adults. Aging Clin Exp Res.

[50] Wildenbos GA, Peute L, Jaspers M (2018). Aging barriers influencing mobile health usability for older adults: a literature based framework (MOLD-US). Int J Med Inform.

[51] Blass D, Rye R, Robbins B, Miner MM, Handel S, Carroll JL, Rabins P (2006). Ethical issues in mobile psychiatric treatment with homebound elderly patients: the Psychogeriatric Assessment and Treatment in City Housing experience. J Am Geriatr Soc.

[52] Bostrom J, Sweeney G, Whiteson J, Dodson JA (2020). Mobile health and cardiac rehabilitation in older adults. Clin Cardiol.

[53] Christensen LF, Moller AM, Hansen JP, Nielsen CT, Gildberg FA (2020). Patients' and providers' experiences with video consultations used in the treatment of older patients with unipolar depression: a systematic review. J Psychiatr Ment Health Nurs.

[54] Gilbert BJ, Goodman E, Chadda A, Hatfield D, Forman DE, Panch T (2015). The role of mobile health in elderly populations. Curr Geri Rep.

[55] Henriquez-Camacho C, Losa J, Miranda JJ, Cheyne NE (2014). Addressing healthy aging populations in developing countries: unlocking the opportunity of eHealth and mHealth. Emerg Themes Epidemiol.

[56] Harerimana B, Forchuk C, O'Regan T (2019). The use of technology for mental healthcare delivery among older adults with depressive symptoms: a systematic literature review. Int J Ment Health Nurs.

[57] Jimison H, Gorman P, Woods S, Nygren P, Walker M, Norris S, Hersh W (2008). Barriers and drivers of health information technology use for the elderly, chronically ill, and underserved. Evid Rep Technol Assess (Full Rep).

[58] Matthew-Maich N, Harris L, Ploeg J, Markle-Reid M, Valaitis R, Ibrahim S, Gafni A, Isaacs S (2016). Designing, implementing, and evaluating mobile health technologies for managing chronic conditions in older adults: a scoping review. JMIR Mhealth Uhealth.

[59] D'Haeseleer I, Gerling K, Vanrumste B, Schreurs D, Buckingham C, Vero VA (2019). Uses and attitudes of old and oldest adults towards self-monitoring health systems. Proceedings of the 13th EAI International Conference on Pervasive Computing Technologies for Healthcare.

[60] Hosseinpour SA, Delavar MR, Hasani Baferani H (2019). A web-based smart telecare system for early diagnosis of heart attack. Int Arch Photogramm Remote Sens Spatial Inf Sci.

[61] Lorenz A, Mielke D, Oppermann R, Zahl L (2007). Personalised mobile health monitoring for elderly. Proceedings of the 9th international conference on Human computer interaction with mobile devices and services.

[62] Pikna J, Fellnerova N, Kozubik M (2018). Information technology and seniors. CBU Int Conf Proc.

[63] Razavi Termeh V, Sadeghi Niaraki A (2015). Design and implementation of ubiquitous health system (u-health) using smart-watches sensors. Int Arch Photogramm Remote Sens Spatial Inf Sci.

[64] Wang X, Knearem T, Carroll J (2018). Learning flows: understanding how older adults adopt and use mobile technology. Proceedings of the 12th EAI International Conference on Pervasive Computing Technologies for Healthcare.

[65] McLoughlin C, Lee MJ (2010). Personalised and self regulated learning in the Web 2.0 era: international exemplars of innovative pedagogy using social software. Aust J Educ Technol.

[66] Ancker JS, Witteman HO, Hafeez B, Provencher T, Van de Graaf M, Wei E (2015). "You Get Reminded You're a Sick Person": personal data tracking and patients with multiple chronic conditions. J Med Internet Res.

[67] Grindrod KA, Li M, Gates A (2014). Evaluating user perceptions of mobile medication management applications with older adults: a usability study. JMIR Mhealth Uhealth.

[68] Fozard J, Wahl H (2012). Age and cohort effects in gerontechnology: a reconsideration. Gerontechnol.

[69] Lim CS (2009). Designing inclusive ICT products for older users: taking into account the technology generation effect. J Eng Design.

[70] Mitsuhashi T (2018). Effects of two-week e-learning on eHealth literacy: a randomized controlled trial of Japanese internet users. PeerJ.

[71] Xie B (2011). Effects of an eHealth literacy intervention for older adults. J Med Internet Res.

[72] Nilsen W, Kumar S, Shar A, Varoquiers C, Wiley T, Riley WT, Pavel M, Atienza AA (2012). Advancing the science of mHealth. J Health Commun.

[73] Chen K, Chan A (2011). A review of technology acceptance by older adults. Gerontechnol.

[74] Kruse CS, Krowski N, Rodriguez B, Tran L, Vela J, Brooks M (2017). Telehealth and patient satisfaction: a systematic review and narrative analysis. BMJ Open.

[75] Doarn C, Shore J, Ferguson S, Jordan P, Saiki S, Poropatich R (2012). Challenges, solutions, and best practices in telemental health service delivery across the pacific rim-a summary. Telemed J E Health.

[76] Jameson J, Farmer M, Head K, Fortney J, Teal C (2011). VA community mental health service providers' utilization of and attitudes toward telemental health care: the gatekeeper's perspective. J Rural Health.

[77] Richardson LK, Frueh BC, Grubaugh AL, Egede L, Elhai JD (2009). Current directions in videoconferencing tele-mental health research. Clin Psychol (New York).

[78] Ricci RP, Morichelli L, Santini M (2009). Remote control of implanted devices through Home Monitoring technology improves detection and clinical management of atrial fibrillation. Europace.

[79] Czaja SJ, Charness N, Fisk AD, Hertzog C, Nair SN, Rogers WA, Sharit J (2006). Factors predicting the use of technology: findings from the Center for Research and Education on Aging and Technology Enhancement (CREATE). Psychol Aging.

[80] Smelcer J, Miller-Jacobs H, Kantrovich L (2009). Usability of electronic medical records. J Usability Stud.

[81] Jutel A (2019). 'The expertness of his healer': diagnosis, disclosure and the power of a profession. Health (London).

[82] Makai P, Perry M, Robben SH, Schers HJ, Heinen MM, Olde Rikkert MG, Melis RF (2014). Evaluation of an eHealth intervention in chronic care for frail older people: why adherence is the first target. J Med Internet Res.

[83] Hallsworth M, Berry D, Sanders M, Sallis A, King D, Vlaev I, Darzi A (2015). Stating appointment costs in SMS reminders reduces missed hospital appointments: findings from two randomised controlled trials. PLoS One.

[84] Landolina M, Perego GB, Lunati M, Curnis A, Guenzati G, Vicentini A, Parati G, Borghi G, Zanaboni P, Valsecchi S, Marzegalli M (2012). Remote monitoring reduces healthcare use and improves quality of care in heart failure patients with implantable defibrillators: the evolution of management strategies of heart failure patients with implantable defibrillators (EVOLVO) study. Circulation.

[85] (2018). The demography of aging in low-and middle-income countries: chronological versus functional perspectives. Future Directions for the Demography of Aging: Proceedings of a Workshop.

[86] Nyella E, Mndeme M (2017). Power tensions in health information system integration in developing countries: the need for distributed control. Electronic J Inf Syst Develop Countries.

