# Supplementary material for: Exploring Barriers to and Enablers of the Adoption of Information and Communication Technology for the Care of Older Adults With Chronic Diseases: Scoping Review
Source: JMIR Aging. 2022 Jan 7;5(1):e25251. doi: 10.2196/25251 (PMC8783284; doi:10.2196/25251)
Supplement: Multimedia Appendix 1 [file aging_v5i1e25251_app1.docx]

Multimedia Appendix 1. Search strategies for Ovid MEDLINE, Embase, Scopus, and PsycINFO databases

**Database 1: Ovid MEDLINE (n=39)**

| Sl. | Searches | Results |
| --- | --- | --- |
| 1 | (information and communication technology).mp. [mp=title, abstract, original title, name of substance word, subject heading word, floating sub-heading word, keyword heading word, organism supplementary concept word, protocol supplementary concept word, rare disease supplementary concept word, unique identifier, synonyms] | 1272 |
| 2 | m-health.mp. [mp=title, abstract, original title, name of substance word, subject heading word, floating sub-heading word, keyword heading word, organism supplementary concept word, protocol supplementary concept word, rare disease supplementary concept word, unique identifier, synonyms] | 397 |
| 3 | mobile health.mp. [mp=title, abstract, original title, name of substance word, subject heading word, floating sub-heading word, keyword heading word, organism supplementary concept word, protocol supplementary concept word, rare disease supplementary concept word, unique identifier, synonyms] | 5969 |
| 4 | e-health.mp. [mp=title, abstract, original title, name of substance word, subject heading word, floating sub-heading word, keyword heading word, organism supplementary concept word, protocol supplementary concept word, rare disease supplementary concept word, unique identifier, synonyms] | 2189 |
| 5 | remote monitoring.mp. [mp=title, abstract, original title, name of substance word, subject heading word, floating sub-heading word, keyword heading word, organism supplementary concept word, protocol supplementary concept word, rare disease supplementary concept word, unique identifier, synonyms] | 1579 |
| 6 | Clinical decision support system.mp. [mp=title, abstract, original title, name of substance word, subject heading word, floating sub-heading word, keyword heading word, organism supplementary concept word, protocol supplementary concept word, rare disease supplementary concept word, unique identifier, synonyms] | 814 |
| 7 | mobile phone technology.mp. [mp=title, abstract, original title, name of substance word, subject heading word, floating sub-heading word, keyword heading word, organism supplementary concept word, protocol supplementary concept word, rare disease supplementary concept word, unique identifier, synonyms] | 178 |
| 8 | telehealth.mp. [mp=title, abstract, original title, name of substance word, subject heading word, floating sub-heading word, keyword heading word, organism supplementary concept word, protocol supplementary concept word, rare disease supplementary concept word, unique identifier, synonyms] | 3631 |
| 9 | electronic health records.mp. [mp=title, abstract, original title, name of substance word, subject heading word, floating sub-heading word, keyword heading word, organism supplementary concept word, protocol supplementary concept word, rare disease supplementary concept word, unique identifier, synonyms] | 22423 |
| 10 | 1 or 2 or 3 or 4 or 5 or 6 or 7 or 8 or 9 | 36996 |
| 11 | ageing.mp. [mp=title, abstract, original title, name of substance word, subject heading word, floating sub-heading word, keyword heading word, organism supplementary concept word, protocol supplementary concept word, rare disease supplementary concept word, unique identifier, synonyms] | 36510 |
| 12 | elderly.mp. [mp=title, abstract, original title, name of substance word, subject heading word, floating sub-heading word, keyword heading word, organism supplementary concept word, protocol supplementary concept word, rare disease supplementary concept word, unique identifier, synonyms] | 225528 |
| 13 | older adults.mp. [mp=title, abstract, original title, name of substance word, subject heading word, floating sub-heading word, keyword heading word, organism supplementary concept word, protocol supplementary concept word, rare disease supplementary concept word, unique identifier, synonyms] | 61556 |
| 14 | 60+ age group.mp. [mp=title, abstract, original title, name of substance word, subject heading word, floating sub-heading word, keyword heading word, organism supplementary concept word, protocol supplementary concept word, rare disease supplementary concept word, unique identifier, synonyms] | 162 |
| 15 | 11 or 12 or 13 or 14 | 305419 |
| 16 | barriers.mp. [mp=title, abstract, original title, name of substance word, subject heading word, floating sub-heading word, keyword heading word, organism supplementary concept word, protocol supplementary concept word, rare disease supplementary concept word, unique identifier, synonyms] | 108381 |
| 17 | enablers.mp. [mp=title, abstract, original title, name of substance word, subject heading word, floating sub-heading word, keyword heading word, organism supplementary concept word, protocol supplementary concept word, rare disease supplementary concept word, unique identifier, synonyms] | 1847 |
| 18 | challenges.mp. [mp=title, abstract, original title, name of substance word, subject heading word, floating sub-heading word, keyword heading word, organism supplementary concept word, protocol supplementary concept word, rare disease supplementary concept word, unique identifier, synonyms] | 215486 |
| 19 | opportunities.mp. [mp=title, abstract, original title, name of substance word, subject heading word, floating sub-heading word, keyword heading word, organism supplementary concept word, protocol supplementary concept word, rare disease supplementary concept word, unique identifier, synonyms] | 103217 |
| 20 | benefits.mp. [mp=title, abstract, original title, name of substance word, subject heading word, floating sub-heading word, keyword heading word, organism supplementary concept word, protocol supplementary concept word, rare disease supplementary concept word, unique identifier, synonyms] | 272838 |
| 21 | threats.mp. [mp=title, abstract, original title, name of substance word, subject heading word, floating sub-heading word, keyword heading word, organism supplementary concept word, protocol supplementary concept word, rare disease supplementary concept word, unique identifier, synonyms] | 17115 |
| 22 | 16 or 17 or 18 or 19 or 20 or 21 | 661550 |
| 23 | arthritis.mp. [mp=title, abstract, original title, name of substance word, subject heading word, floating sub-heading word, keyword heading word, organism supplementary concept word, protocol supplementary concept word, rare disease supplementary concept word, unique identifier, synonyms] | 201917 |
| 24 | asthma.mp. [mp=title, abstract, original title, name of substance word, subject heading word, floating sub-heading word, keyword heading word, organism supplementary concept word, protocol supplementary concept word, rare disease supplementary concept word, unique identifier, synonyms] | 160314 |
| 25 | back pain.mp. [mp=title, abstract, original title, name of substance word, subject heading word, floating sub-heading word, keyword heading word, organism supplementary concept word, protocol supplementary concept word, rare disease supplementary concept word, unique identifier, synonyms] | 53515 |
| 26 | carcinoma.mp. [mp=title, abstract, original title, name of substance word, subject heading word, floating sub-heading word, keyword heading word, organism supplementary concept word, protocol supplementary concept word, rare disease supplementary concept word, unique identifier, synonyms] | 755849 |
| 27 | cardiovascular disease.mp. [mp=title, abstract, original title, name of substance word, subject heading word, floating sub-heading word, keyword heading word, organism supplementary concept word, protocol supplementary concept word, rare disease supplementary concept word, unique identifier, synonyms] | 112867 |
| 28 | chronic obstructive pulmonary disease.mp. [mp=title, abstract, original title, name of substance word, subject heading word, floating sub-heading word, keyword heading word, organism supplementary concept word, protocol supplementary concept word, rare disease supplementary concept word, unique identifier, synonyms] | 41440 |
| 29 | diabetes.mp. [mp=title, abstract, original title, name of substance word, subject heading word, floating sub-heading word, keyword heading word, organism supplementary concept word, protocol supplementary concept word, rare disease supplementary concept word, unique identifier, synonyms] | 545552 |
| 30 | mental health.mp. [mp=title, abstract, original title, name of substance word, subject heading word, floating sub-heading word, keyword heading word, organism supplementary concept word, protocol supplementary concept word, rare disease supplementary concept word, unique identifier, synonyms] | 163305 |
| 31 | non communicable diseases.mp. [mp=title, abstract, original title, name of substance word, subject heading word, floating sub-heading word, keyword heading word, organism supplementary concept word, protocol supplementary concept word, rare disease supplementary concept word, unique identifier, synonyms] | 4404 |
| 32 | chronic diseases.mp. [mp=title, abstract, original title, name of substance word, subject heading word, floating sub-heading word, keyword heading word, organism supplementary concept word, protocol supplementary concept word, rare disease supplementary concept word, unique identifier, synonyms] | 28268 |
| 33 | 23 or 24 or 25 or 26 or 27 or 28 or 29 or 30 or 31 or 32 | 1985578 |
| 34 | 10 and 15 and 22 and 33 | 41 |
| 35 | limit 34 to (English language and humans and yr="2000 -Current") | 39 |

**Database 2: Embase (n= 104)**

|  |
| --- |

| Sl. | Searches | Results |
| --- | --- | --- |
| 1 | (information and communication technology).mp. [mp=title, abstract, heading word, drug trade name, original title, device manufacturer, drug manufacturer, device trade name, keyword, floating subheading word, candidate term word] | 2201 |
| 2 | m-health.mp. [mp=title, abstract, heading word, drug trade name, original title, device manufacturer, drug manufacturer, device trade name, keyword, floating subheading word, candidate term word] | 743 |
| 3 | mobile health.mp. [mp=title, abstract, heading word, drug trade name, original title, device manufacturer, drug manufacturer, device trade name, keyword, floating subheading word, candidate term word] | 4960 |
| 4 | e-health.mp. [mp=title, abstract, heading word, drug trade name, original title, device manufacturer, drug manufacturer, device trade name, keyword, floating subheading word, candidate term word] | 3887 |
| 5 | remote monitoring.mp. [mp=title, abstract, heading word, drug trade name, original title, device manufacturer, drug manufacturer, device trade name, keyword, floating subheading word, candidate term word] | 3921 |
| 6 | Clinical decision support system.mp. [mp=title, abstract, heading word, drug trade name, original title, device manufacturer, drug manufacturer, device trade name, keyword, floating subheading word, candidate term word] | 3868 |
| 7 | mobile phone technology.mp. [mp=title, abstract, heading word, drug trade name, original title, device manufacturer, drug manufacturer, device trade name, keyword, floating subheading word, candidate term word] | 323 |
| 8 | telehealth.mp. [mp=title, abstract, heading word, drug trade name, original title, device manufacturer, drug manufacturer, device trade name, keyword, floating subheading word, candidate term word] | 10271 |
| 9 | electronic health records.mp. [mp=title, abstract, heading word, drug trade name, original title, device manufacturer, drug manufacturer, device trade name, keyword, floating subheading word, candidate term word] | 12973 |
| 10 | 1 or 2 or 3 or 4 or 5 or 6 or 7 or 8 or 9 | 39588 |
| 11 | ageing.mp. [mp=title, abstract, heading word, drug trade name, original title, device manufacturer, drug manufacturer, device trade name, keyword, floating subheading word, candidate term word] | 62386 |
| 12 | elderly.mp. [mp=title, abstract, heading word, drug trade name, original title, device manufacturer, drug manufacturer, device trade name, keyword, floating subheading word, candidate term word] | 562371 |
| 13 | older adults.mp. [mp=title, abstract, heading word, drug trade name, original title, device manufacturer, drug manufacturer, device trade name, keyword, floating subheading word, candidate term word] | 97793 |
| 14 | 60+ age group.mp. [mp=title, abstract, heading word, drug trade name, original title, device manufacturer, drug manufacturer, device trade name, keyword, floating subheading word, candidate term word] | 383 |
| 15 | 11 or 12 or 13 or 14 | 679849 |
| 16 | barriers.mp. [mp=title, abstract, heading word, drug trade name, original title, device manufacturer, drug manufacturer, device trade name, keyword, floating subheading word, candidate term word] | 169948 |
| 17 | enablers.mp. [mp=title, abstract, heading word, drug trade name, original title, device manufacturer, drug manufacturer, device trade name, keyword, floating subheading word, candidate term word] | 3166 |
| 18 | challenges.mp. [mp=title, abstract, heading word, drug trade name, original title, device manufacturer, drug manufacturer, device trade name, keyword, floating subheading word, candidate term word] | 348786 |
| 19 | opportunities.mp. [mp=title, abstract, heading word, drug trade name, original title, device manufacturer, drug manufacturer, device trade name, keyword, floating subheading word, candidate term word] | 164698 |
| 20 | benefits.mp. [mp=title, abstract, heading word, drug trade name, original title, device manufacturer, drug manufacturer, device trade name, keyword, floating subheading word, candidate term word] | 418957 |
| 21 | threats.mp. [mp=title, abstract, heading word, drug trade name, original title, device manufacturer, drug manufacturer, device trade name, keyword, floating subheading word, candidate term word] | 24325 |
| 22 | 16 or 17 or 18 or 19 or 20 or 21 | 1035899 |
| 23 | arthritis.mp. [mp=title, abstract, heading word, drug trade name, original title, device manufacturer, drug manufacturer, device trade name, keyword, floating subheading word, candidate term word] | 369269 |
| 24 | asthma.mp. [mp=title, abstract, heading word, drug trade name, original title, device manufacturer, drug manufacturer, device trade name, keyword, floating subheading word, candidate term word] | 306914 |
| 25 | back pain.mp. [mp=title, abstract, heading word, drug trade name, original title, device manufacturer, drug manufacturer, device trade name, keyword, floating subheading word, candidate term word] | 93409 |
| 26 | carcinoma.mp. [mp=title, abstract, heading word, drug trade name, original title, device manufacturer, drug manufacturer, device trade name, keyword, floating subheading word, candidate term word] | 1256638 |
| 27 | cardiovascular disease.mp. [mp=title, abstract, heading word, drug trade name, original title, device manufacturer, drug manufacturer, device trade name, keyword, floating subheading word, candidate term word] | 370897 |
| 28 | chronic obstructive pulmonary disease.mp. [mp=title, abstract, heading word, drug trade name, original title, device manufacturer, drug manufacturer, device trade name, keyword, floating subheading word, candidate term word] | 72053 |
| 29 | diabetes.mp. [mp=title, abstract, heading word, drug trade name, original title, device manufacturer, drug manufacturer, device trade name, keyword, floating subheading word, candidate term word] | 1098864 |
| 30 | mental health.mp. [mp=title, abstract, heading word, drug trade name, original title, device manufacturer, drug manufacturer, device trade name, keyword, floating subheading word, candidate term word] | 297266 |
| 31 | non communicable diseases.mp. [mp=title, abstract, heading word, drug trade name, original title, device manufacturer, drug manufacturer, device trade name, keyword, floating subheading word, candidate term word] | 7542 |
| 32 | chronic diseases.mp. [mp=title, abstract, heading word, drug trade name, original title, device manufacturer, drug manufacturer, device trade name, keyword, floating subheading word, candidate term word] | 48700 |
| 33 | 23 or 24 or 25 or 26 or 27 or 28 or 29 or 30 or 31 or 32 | 3670267 |
| 34 | 10 and 15 and 22 and 33 | 111 |
| 35 | limit 34 to (English language and humans and yr="2000 -Current") | 104 |

**Database 3: Scopus (n=120)**

| ( ALL ( ( *information*  AND  *communication*  AND *technology* )  OR  ( *m-health* )  OR  ( *mobile*  AND *health* )  OR  ( *telehealth* )  OR  ( *e-health* )  OR  ( *remote*  AND *monitoring* )  OR  ( *clinical*  AND *decision*  AND *support*  AND *system* )  OR  ( *mobile*  AND *phone*  AND *technology* )  OR  ( *electronic*  AND *health*  AND *record* ) )  AND  ALL ( *arthritis*  OR  *asthma*  OR  *back*  AND *pain*  OR  *carcinoma*  OR  *cardiovascular*  AND *disease*  OR  *chronic*  AND *obstructive*  AND *pulmonary*  AND *disease*  OR  *diabetes*  OR  *mental*  AND *health*  OR  *non*  AND *communicable*  AND *diseases*  OR  *chronic*  AND *diseases* )  AND  ALL ( ( *ageing* )  OR  ( *elderly* )  OR  ( *older*  AND *adults* )  OR  ( *60+*  AND *age*  AND *group* ) )  AND  ALL ( *barriers*  OR  *enablers*  OR  *challenges*  OR  *opportunities*  OR  *benefits*  OR  *threats* ) )  AND  DOCTYPE ( *ar*  OR  *re* )  AND  PUBYEAR  >  *1999* |
| --- |

**Database 4: PsycINFO (n=23)**

| Sl. | Searches | Results |
| --- | --- | --- |
| 1 | (information and communication technology).mp. [mp=title, abstract, heading word, table of contents, key concepts, original title, tests & measures, mesh] | 10323 |
| 2 | m-health.mp. [mp=title, abstract, heading word, table of contents, key concepts, original title, tests & measures, mesh] | 135 |
| 3 | mobile health.mp. [mp=title, abstract, heading word, table of contents, key concepts, original title, tests & measures, mesh] | 1277 |
| 4 | e-health.mp. [mp=title, abstract, heading word, table of contents, key concepts, original title, tests & measures, mesh] | 976 |
| 5 | remote monitoring.mp. [mp=title, abstract, heading word, table of contents, key concepts, original title, tests & measures, mesh] | 169 |
| 6 | Clinical decision support system.mp. [mp=title, abstract, heading word, table of contents, key concepts, original title, tests & measures, mesh] | 111 |
| 7 | mobile phone technology.mp. [mp=title, abstract, heading word, table of contents, key concepts, original title, tests & measures, mesh] | 82 |
| 8 | telehealth.mp. [mp=title, abstract, heading word, table of contents, key concepts, original title, tests & measures, mesh] | 1698 |
| 9 | electronic health records.mp. [mp=title, abstract, heading word, table of contents, key concepts, original title, tests & measures, mesh] | 2032 |
| 10 | 1 or 2 or 3 or 4 or 5 or 6 or 7 or 8 or 9 | 16013 |
| 11 | ageing.mp. [mp=title, abstract, heading word, table of contents, key concepts, original title, tests & measures, mesh] | 9894 |
| 12 | elderly.mp. [mp=title, abstract, heading word, table of contents, key concepts, original title, tests & measures, mesh] | 64856 |
| 13 | older adults.mp. [mp=title, abstract, heading word, table of contents, key concepts, original title, tests & measures, mesh] | 48151 |
| 14 | 60+ age group.mp. [mp=title, abstract, heading word, table of contents, key concepts, original title, tests & measures, mesh] | 14 |
| 15 | 11 or 12 or 13 or 14 | 112426 |
| 16 | barriers.mp. [mp=title, abstract, heading word, table of contents, key concepts, original title, tests & measures, mesh] | 59847 |
| 17 | enablers.mp. [mp=title, abstract, heading word, table of contents, key concepts, original title, tests & measures, mesh] | 1317 |
| 18 | challenges.mp. [mp=title, abstract, heading word, table of contents, key concepts, original title, tests & measures, mesh] | 127714 |
| 19 | opportunities.mp. [mp=title, abstract, heading word, table of contents, key concepts, original title, tests & measures, mesh] | 79022 |
| 20 | benefits.mp. [mp=title, abstract, heading word, table of contents, key concepts, original title, tests & measures, mesh] | 107133 |
| 21 | threats.mp. [mp=title, abstract, heading word, table of contents, key concepts, original title, tests & measures, mesh] | 14278 |
| 22 | 16 or 17 or 18 or 19 or 20 or 21 | 347616 |
| 23 | arthritis.mp. [mp=title, abstract, heading word, table of contents, key concepts, original title, tests & measures, mesh] | 6871 |
| 24 | asthma.mp. [mp=title, abstract, heading word, table of contents, key concepts, original title, tests & measures, mesh] | 8004 |
| 25 | back pain.mp. [mp=title, abstract, heading word, table of contents, key concepts, original title, tests & measures, mesh] | 6169 |
| 26 | carcinoma.mp. [mp=title, abstract, heading word, table of contents, key concepts, original title, tests & measures, mesh] | 1922 |
| 27 | cardiovascular disease.mp. [mp=title, abstract, heading word, table of contents, key concepts, original title, tests & measures, mesh] | 9871 |
| 28 | chronic obstructive pulmonary disease.mp. [mp=title, abstract, heading word, table of contents, key concepts, original title, tests & measures, mesh] | 2341 |
| 29 | diabetes.mp. [mp=title, abstract, heading word, table of contents, key concepts, original title, tests & measures, mesh] | 30614 |
| 30 | mental health.mp. [mp=title, abstract, heading word, table of contents, key concepts, original title, tests & measures, mesh] | 219407 |
| 31 | non communicable diseases.mp. [mp=title, abstract, heading word, table of contents, key concepts, original title, tests & measures, mesh] | 568 |
| 32 | chronic diseases.mp. [mp=title, abstract, heading word, table of contents, key concepts, original title, tests & measures, mesh] | 5394 |
| 33 | 23 or 24 or 25 or 26 or 27 or 28 or 29 or 30 or 31 or 32 | 280447 |
| 34 | 10 and 15 and 22 and 33 | 26 |
| 35 | limit 34 to (english language and humans and yr="2000 -Current") [Limit not valid in APA PsycInfo; records were retained] | 23 |
