# Supplementary material for: Exploring Barriers to and Enablers of the Adoption of Information and Communication Technology for the Care of Older Adults With Chronic Diseases: Scoping Review
Source: JMIR Aging. 2022 Jan 7;5(1):e25251. doi: 10.2196/25251 (PMC8783284; doi:10.2196/25251)
Supplement: Multimedia Appendix 2 [file aging_v5i1e25251_app2.docx]

Table 2: Framework analysis of *strengths, weaknesses, opportunities, and threats* on the use of information and communication technology in healthcare among the older adults

| SWOT | Perspectives | Items | Barron et al. | Bhattarai et al. | Chang et al. | Coley et al. | Kim et al. | Zettel-Watson et al. | Lee et al. | Miguel et al. | Mirza et al. | Nymberg et al. | Radhakrishnan et alal. | Rocha et al. | Searcy et al. | Peek et al. | Hutten et al. | Wildenbos et al. | Blass et al. | Bostrom et al. | Christensen et al. | Gilbert et al. | Camacho et al. | Harerimana et al. | Barbera et al. | Matthew et al. | Jimison H et al. | Hosseinpour SA et al. | Termeh VR et al. | Pikna J et al. | Wang Z et al. | Lorenz A et al. | D’Haeseleer et al. |
| --- | --- | --- | --- | --- | --- | --- | --- | --- | --- | --- | --- | --- | --- | --- | --- | --- | --- | --- | --- | --- | --- | --- | --- | --- | --- | --- | --- | --- | --- | --- | --- | --- | --- |
| **Strengths** | Patient-related factors (operational) | Use of an internet platform was a fun and effective way to improve health |  |  |  | x |  |  |  |  | x |  |  | x | x | x |  |  |  | x | x | x | x | x |  |  |  |  |  | x | x | x | x |
|  |  | Satisfaction in using the ICT tools |  |  |  |  |  | x | x | x | x |  | x | x | x | x | x |  |  | x |  |  | x | x |  |  | x |  | x | x | x |  | x |
|  |  | Less face-to-face interaction with clinical staffs, and other patients |  | x |  | x |  | x | x | x | x | x | x |  |  | x |  |  |  | x | x | x | x |  | x | x | x | x |  | x | x | x | x |
|  |  | Reassurance and peace of mind |  |  |  |  |  |  |  | x | x | x | x |  | x | x |  |  | x | x |  |  |  |  | x | x | x |  | x | x | x | x | x |
|  |  | Improved sense of security and reduced anxiety |  |  |  |  |  |  |  | x | x |  | x |  | x | x |  |  | x | x |  | x | x |  |  |  | x | x | x | x | x | x | x |
|  |  | Supported in smoking cessation, physical activity, and healthy diet |  | x |  |  |  |  |  |  | x | x | x | x | x |  | x |  |  | x |  |  |  |  |  | x | x |  |  | x | x | x |  |
|  |  | Increased motivation in doing self-management tasks |  |  | x |  |  |  | x |  | x | x | x |  | x |  |  | x | x | x |  | x | x | x |  |  | x |  | x | x |  | x | x |
|  |  | Prevented functional dependency |  |  | x | x |  |  |  | x | x |  | x | x |  | x |  | x | x |  |  | x | x |  | x | x | x |  | x | x |  | x | x |
|  |  | Benefitted from additional medical monitoring |  |  |  |  | x |  | x | x | x | x | x |  | x |  |  | x |  | x | x | x | x |  |  | x | x | x | x | x |  | x | x |
|  | Healthcare providers- related  factors | Non-pharmacologic nature of the ICT intervention |  |  |  | x |  |  | x |  | x | x | x | x |  | x |  |  | x | x |  | x | x | x | x |  | x |  | x | x |  | x |  |
|  |  | An interactive push-notification feature |  |  | x |  |  |  |  |  | x |  |  | x | x | x | x | x |  |  |  | x | x | x |  |  | x | x |  | x |  |  | x |
|  |  | Larger screens were user friendly | x |  |  |  |  |  |  | x |  |  |  |  |  | x |  |  |  | x |  |  |  |  |  | x |  |  |  |  |  | x |  |
|  |  | Written instructions were helpful | x |  |  |  |  |  |  |  |  |  |  |  |  | x |  | x |  |  |  |  |  |  |  |  |  |  |  |  |  |  |  |
|  |  | Desire to see an increase in available functions |  |  |  |  |  | x |  |  |  |  |  | x | x | x |  | x |  | x |  | x | x |  |  |  |  |  |  |  |  | x |  |
| **Weaknesses** | Patient-related factors (operational) | Lack of confidence in computer skills |  |  |  |  | x |  |  |  |  | x |  |  | x | x |  | x |  |  |  | x | x | x |  |  |  |  |  |  | x | x | x |
|  |  | The inconvenience of having continuous access to an internet |  |  |  | x |  |  |  |  | x | x | x |  |  | x |  | x |  |  | x | x |  |  | x |  | x |  | x | x | x |  |  |
|  |  | Required support (supervisor) in maintaining disease management behaviors |  |  | x |  |  |  | x | x |  |  |  |  |  |  |  | x |  |  | x |  | x | x |  | x |  |  | x | x | x | x | x |
|  |  | Concerned about bothering their family members to operate the equipment |  | x | x |  |  |  |  |  |  |  |  |  |  | x |  | x | x | x |  | x | x |  |  |  |  |  |  |  |  |  | x |
|  |  | Participants did not voluntarily learn to use the equipment if their family members were capable of operating it for them |  |  | x |  |  |  | x |  |  |  |  |  | x | x |  | x |  | x |  | x | x | x |  |  |  |  |  |  |  | x |  |
|  |  | Felt embarrassed when they fail to correctly operate technology | x |  | x |  |  |  |  |  | x | x | x |  |  | x |  | x |  |  | x |  | x | x |  |  | x |  |  | x | x | x | x |
|  |  | Become concerned with data or privacy |  |  |  |  |  | x |  |  |  | x | x |  |  | x |  |  | x | x |  | x | x |  |  |  |  |  |  |  |  | x |  |
|  | Healthcare providers- related  factors | Technology specific to this age group was usually operated by other family members |  |  | x |  |  |  |  |  |  |  |  |  | x |  |  | x |  | x |  |  |  |  |  |  |  |  |  |  |  | x |  |
|  |  | Small screens and cramped keyboards |  |  |  |  |  |  |  |  | x |  |  | x | x | x | x |  |  | x | x | x | x |  |  |  |  |  |  | x |  |  |  |
|  |  | Medical terms unfamiliar | x |  |  |  |  |  |  |  |  |  |  |  |  | x |  |  |  |  |  |  | x | x |  |  |  |  |  |  | x |  |  |
|  |  | Provider’s Lack of necessary computer skills |  |  |  |  | x |  | x |  |  |  |  |  |  | x | x | x |  |  | x | x | x | x |  |  |  |  |  | x | x |  | x |
|  |  | Found the electronic tool hard to use | x |  |  |  |  | x |  |  |  |  |  |  |  | x |  |  |  | x | x | x | x |  |  |  | x |  |  | x | x | x | x |
|  |  | Inadequacy of battery power |  |  |  |  |  |  |  |  | x |  |  | x | x | x |  |  |  |  |  |  |  |  |  | x |  |  |  | x | x | x |  |
| **Opportunities** | Patient-related factors (operational) | Regular medical check-ups |  |  |  | x |  |  |  | x | x | x | x | x |  | x |  | x |  |  |  |  | x | x | x |  |  | x | x | x | x | x | x |
|  |  | Benefits of lifestyle changes |  |  |  | x |  |  |  | x | x | x | x |  |  | x |  | x |  | x | x |  | x | x | x | x | x | x | x | x | x |  | x |
|  |  | Initial encouragement from the doctors, and financial incentives compel patients to use ICT services |  |  | x |  |  |  | x |  | x | x | x |  | x | x |  | x |  | x | x | x | x |  |  | x | x |  | x | x | x | x | x |
|  |  | Recommend the tool to others |  |  |  |  |  |  |  |  | x |  | x |  |  | x |  | x |  | x | x | x | x |  |  |  | x |  |  | x |  | x | x |
|  |  | Were partially willing to pay for ICT services |  |  | x |  |  |  |  |  |  |  |  |  |  | x |  | x |  |  | x | x | x | x |  |  |  |  |  |  |  |  |  |
|  |  | Required specific practical advice and encouragement about making lifestyle changes | x |  |  |  | x |  | x | x | x |  | x | x |  | x | x | x |  | x | x |  | x | x |  |  | x |  |  | x | x | x |  |
|  |  | Developed their self-care disease management skills and maintain quality of life |  |  | x |  | x | x | x | x |  | x |  | x | x | x | x | x |  |  | x | x | x |  |  | x |  | x | x |  | x | x | x |
|  |  | An app should have personalisation features to suit the user ’s preferences |  | x |  |  |  |  |  | x |  |  |  | x | x | x | x | x |  |  |  |  | x | x |  |  |  | x |  | x | x | x | x |
|  |  | It should provide local context |  | x |  |  |  |  |  | x |  |  | x |  |  | x |  |  |  | x | x | x | x |  |  |  | x | x |  |  |  | x | x |
|  |  | Simplified and easy-to-use operations |  |  | x |  |  |  |  |  | x | x | x | x | x | x | x |  |  | x | x | x | x | x |  | x | x |  | x | x |  | x | x |
|  |  | Equipment should be portable, and rechargeable |  |  | x |  |  |  |  |  | x |  |  | x | x | x | x |  |  |  | x |  | x | x |  |  |  | x | x | x |  | x | x |
|  | Healthcare providers- related  factors | Clinician's involvement was crucial when integrating an app into routine practices |  | x |  |  |  | x | x | x | x | x | x |  |  |  |  | x | x | x |  |  | x | x |  | x | x |  |  | x | x | x | x |
|  |  | Special care to older adults when cognitive and sensory functions were impaired |  |  | x |  |  |  |  |  | x | x |  | x | x |  | x |  |  | x | x | x | x | x |  | x |  |  |  | x | x | x | x |
| Threats | Patient-related factors (operational) | No intention of learning how to use the technology |  |  | x |  |  |  |  |  |  |  |  |  | x | x |  | x |  | x |  |  |  |  |  |  |  |  |  |  |  |  | x |
|  |  | Perceived new technology as being expensive and complex |  |  | x |  |  |  |  |  |  | x |  | x |  |  |  | x |  |  | x | x | x |  |  | x |  |  |  | x |  |  | x |
|  |  | Most of them suffered from hearing and sight impairment, these can obstruct communication |  |  | x |  |  |  |  |  |  |  |  | x |  |  |  | x |  | x | x |  | x | x | x |  |  |  |  |  |  | x |  |
|  |  | Despite significant improvements in the patients' self-care, they were unwilling to continue ICT services if these are not free of charge | x |  | x |  |  |  |  |  | x |  | x |  |  |  |  | x |  |  | x | x | x | x |  |  | x |  |  | x |  | x |  |
|  |  | No longer have a need for using the tool |  |  |  |  |  | x |  |  | x |  |  |  |  |  |  |  |  |  | x | x | x |  |  |  |  |  |  | x |  |  | x |
|  |  | Breach of confidentialities |  | x |  |  |  |  |  |  |  |  |  |  |  |  |  |  | x |  | x |  |  | x |  |  |  |  |  |  |  |  |  |
|  | Healthcare providers- related  factors | mHealth care should not replace regular health care practices |  |  |  |  | x |  |  |  |  | x |  |  |  |  |  |  | x |  |  | x | x |  | x | x |  | x | x |  |  | x |  |
|  |  | Patients considered not using ICT if their doctors stopped offering the tool |  |  |  |  |  | x |  |  | x |  | x |  |  |  |  | x |  |  | x |  | x | x |  |  | x |  |  | x |  |  | x |

SWOT: Strengths, weaknesses, opportunities, and threats

ICT: Information and communication technology
