# Supplementary material for: Exploring Barriers to and Enablers of the Adoption of Information and Communication Technology for the Care of Older Adults With Chronic Diseases: Scoping Review
Source: JMIR Aging. 2022 Jan 7;5(1):e25251. doi: 10.2196/25251 (PMC8783284; doi:10.2196/25251)
Supplement: Multimedia Appendix 3 [file aging_v5i1e25251_app3.docx]

Multimedia Appendix 3. List of barriers to and challenges for the use of information and communication technology among older adults

| *Domain* | *Types of challenges and barriers* |
| --- | --- |
| For the elderly | |
| Patient motivation | - Patient’s willingness and motivation to manage their health is necessary - Older adults are not as willing to try new methods |
| Presence of comorbidities and additional risk factors | - Older adults with chronic disease often have other comorbidities such as diabetes, obesity, sleep apnea, or mental health disorders. Thus, when wearable devices are used to monitor one or two conditions, additional support may be required for patients to deal with other comorbidities |
| Adherence to treatment following ICT platform intervention | - Ensuring adherence to treatment can be a challenge - An older adult can feel bored in using ICT platforms |
| Lack of prior experience to operate ICT devices | - Older adults should have previous experience in technology, and some sort of orientation, and training - Health professionals also need technology skills |
| ICT Implementation related | |
| Implementation costs | - Implementation costs of devices can be high - In many countries, there is a lack of reimbursement for adopting ICT devices or applications, which imposes additional costs for patients - Integration of various technology |
| The maintenance cost of devices and the internet for patients | - Device cost is a barrier - All areas are not equipped with useful internet or broadband - Not all belonged to a smartphone |
| Context and location-specific | - Only appropriate to use in developed countries - Some ICT tools are not adapted to work on emergency response - Rural hospitals did not implement ICT use as widely as urban hospitals |
| Infrastructure (home setting) | - Often requires the installation of new devices, hardware, and software applications in a home setting of the patient - High bandwidth internet availability may not be suitable for low resource settings |
| Capacity development of the staffs | - Monitoring the continuous flow of data requires skilled personnel and additional workload in the healthcare sector - Time consuming (time is required for staff training) |
| Issues related to data security | - There is a lack of protection to keep the data safe - Lack of confidentiality can be a future challenge in using a medical record of a patient from the database |
| Lack of data standardisation | - Data exchange and interoperability among multiple healthcare providers is difficult to establish - It might be challenging to compare the quality of data across various settings |
| Time to take in making the decision | - The question of liability might affect how fast a physician might be reasonably expected to react in response to transmitted alerts |
| Device maintenance from the provider’s perspective | - Implantable electronic devices require regular maintenance and calibration to ensure technical integrity - Depending on the device and the underlying disease condition, the frequency of follow-up maintenance varies |
| Time-consuming | - Clinicians do not always use electronic health record, as it takes time to use correctly |
| Inadequate quality and safety of ICT services | - Quality and security have to be ensured from the perspective of the use of various machines |

CVD: Cardiovascular disease

ICT: Information and communication technology
